# Molecular stratification of metastatic melanoma using gene expression profiling : Prediction of survival outcome and benefit from molecular targeted therapy

**DOI:** 10.18632/oncotarget.3655

**Published:** 2015-03-26

**Authors:** Helena Cirenajwis, Henrik Ekedahl, Martin Lauss, Katja Harbst, Ana Carneiro, Jens Enoksson, Frida Rosengren, Linda Werner-Hartman, Therese Törngren, Anders Kvist, Erik Fredlund, Pär-Ola Bendahl, Karin Jirström, Lotta Lundgren, Jillian Howlin, Åke Borg, Sofia K. Gruvberger-Saal, Lao H. Saal, Kari Nielsen, Markus Ringnér, Hensin Tsao, Håkan Olsson, Christian Ingvar, Johan Staaf, Göran Jönsson

**Affiliations:** ^1^ Department of Clinical Sciences, Division of Oncology and Pathology, Lund University, Lund, Sweden; ^2^ Department of Clinical Sciences, Division of Surgery, Lund University, Lund, Sweden; ^3^ Department of Oncology, Skåne University Hospital, Lund University, Lund, Sweden; ^4^ Department of Clinical Pathology, Skåne University Hospital, Lund University, Lund, Sweden; ^5^ Department of Oncology-Pathology, Karolinska Institute, Stockholm, Sweden; ^6^ Department of Dermatology, Helsingborg General Hospital, Helsingborg, Sweden; ^7^ Department of Dermatology, Harvard Medical School, Boston, USA; ^8^ Wellman Center for Photomedicine, MGH Cancer Center, Massachusetts General Hospital, Boston, USA

**Keywords:** gene expression, melanoma, BRAF, BRAF inhibitor, mutation

## Abstract

Melanoma is currently divided on a genetic level according to mutational status. However, this classification does not optimally predict prognosis. In prior studies, we have defined gene expression phenotypes (high-immune, pigmentation, proliferative and normal-like), which are predictive of survival outcome as well as informative of biology. Herein, we employed a population-based metastatic melanoma cohort and external cohorts to determine the prognostic and predictive significance of the gene expression phenotypes. We performed expression profiling on 214 cutaneous melanoma tumors and found an increased risk of developing distant metastases in the pigmentation (HR, 1.9; 95% CI, 1.05-3.28; *P*=0.03) and proliferative (HR, 2.8; 95% CI, 1.43-5.57; *P*=0.003) groups as compared to the high-immune response group. Further genetic characterization of melanomas using targeted deep-sequencing revealed similar mutational patterns across these phenotypes. We also used publicly available expression profiling data from melanoma patients treated with targeted or vaccine therapy in order to determine if our signatures predicted therapeutic response. In patients receiving targeted therapy, melanomas resistant to targeted therapy were enriched in the MITF-low proliferative subtype as compared to pre-treatment biopsies (*P*=0.02). In summary, the melanoma gene expression phenotypes are highly predictive of survival outcome and can further help to discriminate patients responding to targeted therapy.

## INTRODUCTION

Cutaneous malignant melanoma (CMM) is the most lethal form of skin cancer and its incidence has increased faster than that of any other cancer, rendering it a major public health problem worldwide. In order to provide clinicians and patients with accurate prognostic information about the disease, a correct staging system is fundamental. Since 1998, the American Joint Committee on Cancer (AJCC) melanoma staging system has served as a foundation for clinical classification and it was recently updated to the 7^th^ edition after adding more tumor intrinsic factors with prognostic significance [1]. However, clinical outcome of patients with similar or even identical clinical and histological features varies considerably [2], especially within the AJCC intermediate risk stages and in patients with advanced disease [3].

Gene expression profiling may provide additional information to the current prognostic assessment. Several attempts to introduce this approach as a step towards individualized patient management have been made by defining new molecular biomarkers and gene signatures correlating with clinical outcome. Since the initial search for prognostic signatures in melanoma by Winnepenninckx et al. [4], several signatures have been proposed. These range from a single-gene signature of osteopontin in primary melanoma, to different multi-gene signatures in stage III and IV metastatic melanoma lesions [5-8]. However, at present time there is still no extensively validated prognostic molecular signature in melanoma.

We have reported distinct gene expression phenotypes significantly associated with survival outcome in stage IV metastatic melanoma [9]. The phenotypes, mainly characterized by differential expression of immune response, melanocyte-specific and proliferation genes, have also been validated in cutaneous primary melanoma [10].

In melanoma, molecular targeted anticancer therapy has received much focus lately and new emerging treatment approaches have shown dramatic clinical results. The prospective drugs include kinase inhibitors, targeting oncogenic BRAFV600E or MEK [11]; immune system activators, e.g. vaccines or immune checkpoint blockades, with the latter approach utilizing monoclonal antibodies directed against the inhibitory immune receptors CTLA-4, PD-1 or PD-L1 [12-14]. Since treatment with these agents can be associated with significant morbidity and since response is far from universal, there is a compelling need to better identify patients who may benefit from the newer generation therapies.

In the present work we aimed to further establish the clinical relevance and delineate the mutational landscape of our previously described gene expression phenotypes in a population-based retrospective collection of 214 CMM specimens obtained from a single clinical institution. This is, to our knowledge, the largest study correlating genome-wide molecular and mutation data to clinical patient information in metastatic melanoma. Our data firmly validates the prognostic significance of the gene expression phenotypes and provides novel evidence that the gene expression phenotypes may predict benefit from molecular targeted therapies in advanced stage melanoma beyond BRAF status.

## RESULTS

### Repeated observation of gene expression phenotypes in a population-based metastatic melanoma cohort

**Figure 1:**
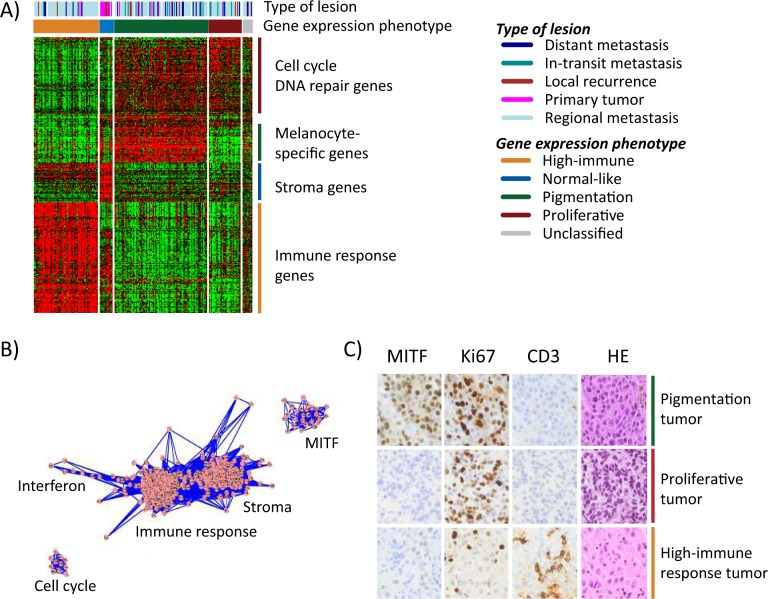
Gene expression phenotypes in melanoma **A**) Heatmap of 299 genes (rows) included in the classification of 214 melanoma tumors (columns). Tumor descriptions are shown in the color bars including phenotype classification and tumor type. **B**) Network analysis identified five clusters of genes reflecting biological mechanisms of relevance in melanoma and named thereafter. Each dot (pink) represents a gene that is connected by lines (blue) representing correlations between the genes. **C**) Immunohistochemical staining of MITF, Ki67, CD3 were performed in 59 tumors. Three representative tumors of the gene expression phenotypes are shown in the figure. Sections were also stained with hematoxylin/eosin (HE) to see structural patterns in the tissues.

Gene expression profiling was performed on a total of 214 CMM tumor tissues representing a population-based retrospective collection from a single institution. In this cohort, all previously reported phenotypes were present: high-immune response (30%), normal-like (6%), pigmentation (44%) and proliferative (15%) (Figure 1A) [9]. An overview of the patient clinicopathological characteristics and the gene expression phenotypes is provided in Table 1. Specifically, the normal-like group was more prevalent among the primary melanoma tumors, comprising 50% (8/16), whereas only 2% (4/188) of the tumors in the metastatic setting were classified as normal-like. When excluding the normal-like group (due to lower number of cases), there was no significant association between gene expression phenotype and metastasis type (*P*=0.1, Fisher's exact test). Furthermore, when analyzing primary melanoma tumor features we found that histological type and primary site varied between the phenotypes (*P*=0.01 or *P*=0.02, respectively, Fisher's exact test). In contrast, Breslow thickness, age at primary diagnosis and Clark's level of invasion did not show any significant differences (*P* > 0.05, Table 1). Analysis of the median time from primary melanoma diagnosis to the diagnosis of the analysed metastatic lesion indicated a significant difference between the groups, with longer durations for the proliferative-classified melanomas (*P*=0.04, Kruskal-Wallis test).

**Table 1:** Clinical characteristics of 214 melanoma patients and their tumors

Clinical parameters	Whole cohort (N=214)	High-immunegroup (n=65)	Normal-likegroup (n=13)	Pigmentationgroup (n=94)	Proliferativegroup (n=32)	Unclassifiedgroup (n=10)	P-value^1*^
***Patient characteristics***							
*Gender, n (%)*							0.2
Male	124 (58)	36 (55)	6 (46)	62 (66)	16 (50)	4 (40)	
Female	89 (42)	29 (45)	7 (54)	31 (33)	16 (50)	6 (60)	
NA	1 (0.5)	0 (0)	0 (0)	1 (1)	0 (0)	0 (0)	
*Age*^2^*, n (%)*							0.1
<60	80 (37)	23 (35)	2 (15)	41 (44)	8 (25)	6 (60)	
≥60	130 (61)	40 (62)	10 (77)	52 (55)	24 (75)	4 (40)	
NA	4 (2)	2 (3)	1 (8)	1 (1)	0 (0)	0 (0)	
***Tumor characteristics***							
*Tumor type, n (%)*							<0.001
Primary	16 (7)	2 (3)	8 (62)	5 (5)	1 (3)	0 (0)	
Metastasis	188 (88)	61 (94)	4 (31)	83 (88)	30 (94)	10 (100)	
NA	10 (5)	2 (3)	1 (8)	6 (6)	1 (3)	0 (0)	
*Metastasis type, n (%)*							0.003
Local	11 (5)	2 (3)	3 (23)	3 (3)	2 (6)	1 (10)	
In-transit	15 (7)	2 (3)	0 (0)	8 (9)	5 (16)	0 (0)	
Regional	139 (65)	52 (80)	1 (8)	58 (62)	21 (66)	7 (70)	
General	23 (11)	5 (8)	0 (0)	14 (15)	2 (6)	2 (20)	
NA^3^	26 (12)	4 (6)	9 (69)	11 (12)	2 (6)	0 (0)	
*No.of somatic mutations^4^. median (range)*	110 (5-768)	91 (5-404)	190 (45-397)	113 (6-768)	115 (18-364)	87 (31-293)	0.5
*MAPK pathway^4,5^, n (%)*							
mut	123 (84)	22 (79)	3 (75)	66 (85)	26 (96)	6 (67)	0.3
wt	23 (16)	6 (21)	1 (25)	12 (15)	1 (4)	3 (33)	
***Primary tumor and patient characteristics***							
*Age*^2^*, n (%)*							0.6
<60	76 (36)	22 (34)	3 (23)	33 (35)	12 (38)	6 (60)	
≥60	95 (44)	30 (46)	9 (69)	40 (43)	13 (41)	3 (30)	
NA	43 (20)	13 (20)	1 (8)	21 (22)	7 (22)	1 (10)	
*Breslow (median, mm)*	2.5	2.6	4.0	2.3	2.5	2.5	0.5
*Clark level, n (%)*							0.4
I	1 (0.5)	0 (0)	0 (0)	0 (0)	1 (3)	0 (0)	
II	6 (3)	3 (5)	1 (8)	1 (1)	0 (0)	1 (10)	
III	45 (21)	12 (18)	2 (15)	22 (23)	7 (22)	2 (20)	
IV	78 (36)	28 (43)	5 (38)	30 (32)	11 (34)	4 (40)	
V	17 (8)	5 (8)	3 (23)	6 (6)	2 (6)	1 (10)	
NA	67 (31)	17 (26)	2 (15)	35 (37)	11 (34)	2 (20)	
*Histologic type, n (%)*							0.01
Unknown primary	28 (13)	9 (14)	0 (0)	13 (14)	5 (16)	1 (10)	
SSM	47 (22)	13 (20)	3 (23)	20 (21)	7 (22)	4 (40)	
NM	72 (34)	25 (38)	1 (8)	29 (31)	14 (44)	3 (30)	
Other^6^	15 (7)	2 (3)	5 (38)	8 (9)	0 (0)	0 (0)	
NA	52 (24)	16 (25)	4 (31)	24 (26)	6 (19)	2 (20)	
*Primary site, n (%)*							0.02
Upper limbs	25 (12)	4 (6)	0 (0)	11 (12)	8 (25)	2 (20)	
Lower limbs	61 (29)	19 (29)	6 (46)	24 (26)	10 (31)	2 (20)	
Trunk	72 (34)	27 (42)	3 (23)	31 (33)	7 (22)	4 (40)	
Other^7^	14 (7)	2 (3)	3 (23)	8 (9)	0 (0)	1 (10)	
NA	42 (20)	13 (20)	1 (8)	20 (21)	7 (22)	1 (10)	
*Ulceration, n (%)*							0.6
Yes	52 (24)	14 (22)	7 (54)	23 (24)	6 (19)	2 (20)	
No	39 (18)	13 (20)	2 (15)	16 (17)	5 (16)	3 (30)	
NA	123 (57)	38 (58)	4 (31)	55 (59)	21 (66)	5 (50)	
*Primary – Metastasis*							
Time (median months, range)^8^	27 (0-461)	25 (0-214)	26 (0-195)	24 (0-229)	46 (3-461)	31 (1-125)	0.04

Abbreviations: NA, not available; SSM, superficial spreading melanoma; NM, nodular melanoma; ALM, acral lentiginous melanoma; LMM, Lentigo maligna melanoma; wt, wild-type; mut, mutation

1 By Fisher's exact test, except for No. of somatic mutations, Breslow thickness, Clark classification and primary-metastasis time (Kruskal-Wallis test).

2 Age at diagnosis/surgery.

3 Primary and NAs.

4 Deep targeted sequencing of 146 samples with following GEX phenotypes: High-immune, 28; Normal, 4; Pigmentation, 78; Proliferative, 27.

5 Analysis of hotspot mutations in: BRAF (V600): NRAS (G12, G13, Q61), NF1 (stopgains): KIT (all mutations).

6 ALM, LMM and other melanoma types.

7 Head/neck and tumors from other anatomical sites.

8 Only patients with disease progression (excluding primary cases).

* Not including unclassified samples and NA information in the analyzes.

To further describe the phenotypes and determine transcriptional programs in melanoma, we performed transcriptional network analysis of highly correlated genes in the cohort, as previously described [15]. Based on gene ontology analysis and published associations with melanoma-specific tumor biology, we could extract five transcriptional modules defined herein as the micropthalmia-associated transcription factor (MITF), cell cycle, stroma, immune response and interferon modules (Figure 1B, Supplementary Table 1). As expected, the high-immune response phenotype was highly associated with the immune response and stroma modules, and less associated with the cell cycle and MITF modules (Figure S1A-B). The pigmentation phenotype was correlated with a high MITF and cell cycle module activity, whereas a lower association was found with the immune response and stroma modules (Figure S1A-B). The proliferative phenotype was associated with a high cell cycle activity but not with any of the other modules (Figure S1A-B). Thus, the transcriptional modules reflect gene expression phenotypes to a large extent.

To investigate whether gene expression phenotypes are reflected on protein expression levels, we examined MITF (a melanocyte-specific marker), cluster of differentiation 3 (CD3, expressed by mature T-lymphocytes), and the proliferative marker Ki67 in a subset of our tumors (*n*=59) by immunohistochemical analysis. A striking agreement between protein and gene expression was observed in the phenotypes with strong infiltration of CD3 positive T lymphocytes in the high-immune response classified tumors and a high prevalence of Ki67 positive melanoma cells in the proliferative tumors with few, if any, cells staining positive for MITF (Figure 1C). The pigmentation-classified tumors comprised a high fraction of MITF positive cells and a high prevalence of Ki67 positive cells (Figure 1C).

### Gene expression phenotypes and somatic mutation status

**Figure 2:**
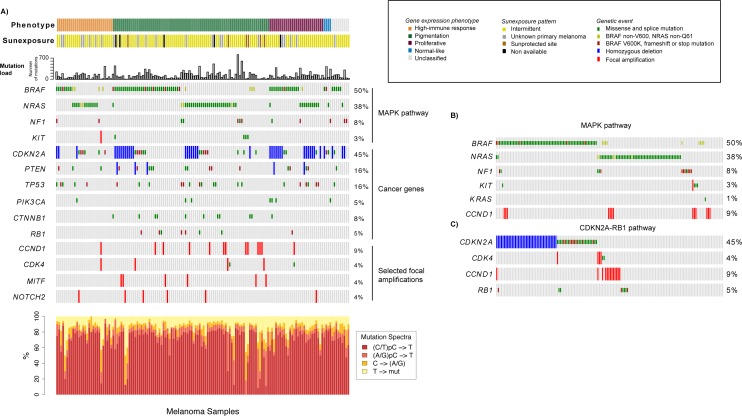
Analysis of the mutational landscape in melanoma tumors **A**) Genetic events such as mutations, homozygous deletions and focal amplifications in cancer genes within the context of the gene expression phenotypes. Tumors are ordered according to the gene expression phenotypes and the genes of interest. The mutation frequency plot corresponds to the number of somatically acquired mutations observed in the 1697 investigated cancer-associated genes in each melanoma tumor. **B**) Mutations in genes involved in the MAPK pathway. Tumors are ordered according to mutations in *BRAF*, *NRAS*, *NF1*, *KIT*, *KRAS* and *CCND1*. **C**). Genetic events in genes involved in the CDKN2A-RB1 pathway. Tumors are ordered according to genetic events in *CDKN2A*, *CDK4*, *CCND1* and *RB1.*

To further characterize the mutational landscape of the gene expression phenotypes, we used targeted deep sequencing to screen for somatic mutations in 1697 cancer-associated genes in tumors from 146 CMM patients. Among these tumors, the mutation burden demonstrated wide heterogeneity, ranging from 5 up to 768 somatic mutations per tumor (Table 1). A small subset of acral lentiginous melanomas (ALMs, *n*=6) had a significantly lower mutation burden (range: 6-51 mutations), as compared to metastases of unknown origin, superficial spreading or nodular melanoma (*P* < 0.001, Kruskal-Wallis test). Moreover, the ALMs were all classified as pigmentation tumors. The mutation burden was not significantly different between the gene expression phenotypes (*P*=0.5, Kruskal-Wallis test) (Table 1). Furthermore, most melanomas harbored the UV-induced mutational signature C -> T preceded by a pyrimidine (Figure 2).

Next, we focused on a set of genes highly implicated in melanomagenesis, including *BRAF*, *NRAS*, *KIT*, *CDK4*, *CDKN2A*, *TP53*, *PTEN*, *CTNNB1*, *NF1*, *RB1* and *PIK3CA*. To provide a more comprehensive context of the mutational landscape we also included focal amplifications and homozygous deletions of melanoma signature genes, like *MITF*, *KIT*, *CDK4*, *NOTCH2* and *CCND1, CDKN2A* and *PTEN* (Figure 2A). Analysis of the mutation spectrum within the context of the gene expression phenotypes showed that: i) *CDKN2A* alterations were more prevalent in the proliferative-classified melanomas (*P*=0.05, Fisher's exact test, consistent with previous reports [9]), ii) pigmentation-classified melanomas were enriched for genetic events in *CTNNB1, MITF* or *CCND1* (*P*=0.02, *P*=0.04 and *P*=0.04, respectively, Fisher's exact test), whereas iii) *BRAF* and *NRAS* mutations were equally distributed across phenotypes (Supplementary Table 2).

*BRAF* and *NRAS* mutations were mutually exclusive in 96% of tumors, while six cases had co-occurring mutations (*P* < 0.001, Fisher's exact test). Interestingly, the majority of the samples with concurrent *BRAF* and *NRAS* mutations (4/6 tumors) seemed to have a *NRAS* hotspot mutation and a non-hotspot mutation of *BRAF* (Figure 2B). The BRAF/NRAS wild-type melanomas, i.e. samples negative for mutations in both *BRAF* and *NRAS*, more often had genetic alterations in *KIT* or *NF1* (*P* < 0.001, Fisher's exact test) (Figure 2B). In total, only 9.6% of all cases in the cohort were negative for either of the genetic changes in *BRAF*, *NRAS*, *NF1* and *KIT*, indicating that the majority of melanomas could potentially have activated MAPK pathway through these genetic alterations. In addition, one tumor negative for these four genes harbored a *KRAS* G13D mutation. When considering hotspot mutations in *BRAF* and *NRAS*, alterations in *KIT* and loss of function mutations in *NF1*, we found that the majority (96%) of the proliferative-classified tumors had an alteration. Furthermore, we also found mutually exclusive genetic events in the CDKN2A-RB1 pathway where *CDK4*, *CCND1* and *RB1* alterations occurred mainly in *CDKN2A* wild-type tumors (Figure 2C). In summary, these results suggest that the most common genetic alterations in melanoma occur at similar frequency, whereas some less frequently mutated genes may be enriched in gene expression phenotypes.

### Gene expression phenotypes are predictive of survival outcome in melanoma

**Figure 3:**
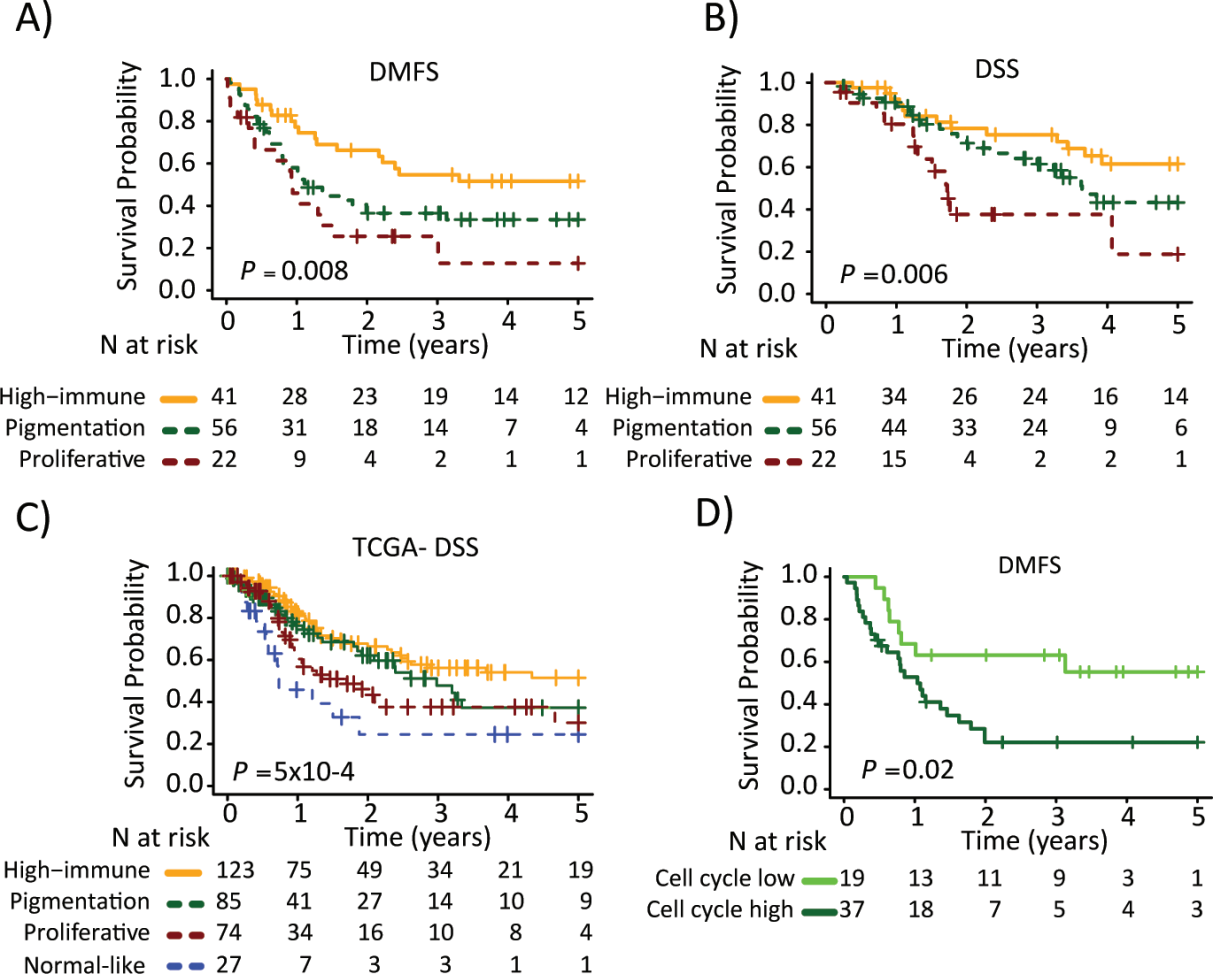
Survival analysis of metastatic melanomas stratified by gene expression phenotype using the Kaplan-Meier estimator to determine **A**) Distant metastasis free survival (DMFS) and **B**) and disease specific survival (DSS). **C**) Metastatic tumors from the TCGA data were stratified and Kaplan-Meier analysis was performed. **D**) Pigmentation-classified tumors were stratified by the cell cycle module (low or high). Survival differences between low and high groups were estimated using Kaplan-Meier analysis. P-values have been calculated using the log-rank test.

Next, we determined the association between the gene expression phenotypes and the survival outcome in the metastatic cohort. Among the patients with regional metastatic disease (*n*=125), we found an increased risk of having a distant metastasis (5-year distant metastasis-free survival, DMFS) in the pigmentation (HR, 1.9; 95% CI, 1.05-3.28; *P*=0.03) and proliferative (HR, 2.8; 95% CI, 1.43-5.57; *P*=0.003) phenotypes, as compared to the high-immune response phenotype (Table 2). In addition, an increased risk of death from melanoma (5-year disease-specific survival, DSS) was observed in the pigmentation (HR, 1.7; 95% CI, 0.83-3.28; *P*=0.2) and proliferative (HR, 3.5; 95% CI, 1.56-7.80; *P*=0.002) phenotypes, as compared to the high-immune response phenotype (Table 2). The corresponding Kaplan-Meier analyzes are shown in Figures 3A and 3B, respectively. In a multivariable Cox regression model (adjusting for age, gender and metastasis type), with the high-immune response phenotype as the reference group, the pigmentation and proliferative phenotypes exhibited an increased risk of distant metastases, whereas only the proliferative phenotype had a significantly increased risk of death from melanoma (Table 2). The confounders were chosen based on their level of significance from the univariable analyzes with a cutoff at *P* ≤ 0.05 (Supplementary Table 3). In the stage IV melanoma cohort, we analyzed the distribution of the gene expression phenotypes among the distantly metastasized tumors (*n*=23). In total, there were two proliferative-classified melanomas and both had a poor survival (Figure S2). To further validate our findings we used the 309 regional and distant metastatic lesions from the TCGA data set. Here, we could firmly validate an improved survival in the high-immune response group as compared to the other groups (*P*=5×10^−4^, Figure 3C).

**Table 2:** Survival outcome analysis in patients with regional metastatic disease: Cox regression analysis of gene expression phenotypes

	Univariable analysis				Multivariable analysis^3^				Confounders^3^
	Events/N	HR	95% CI	P^*^	Events/N	HR	95% CI	P^*^	
***Distant metastasis-free survival^1^***									
*GEX phenotypes^4^*	69/119				69/118				
High-immune		1.00	Ref	(0.01)		1.00	Ref	(0.02)	Gender, age
Pigmentation		1.9	1.05-3.28	0.03		1.8	1.00-3.17	0.05	
Proliferative		2.8	1.43-5.57	0.003		2.7	1.37-5.36	0. 004	
*Gender*	72/125								
Female		1.00	Ref	(0.02)		1.00	Ref	(0.006)	
Male		1.9	1.13-3.17	0.02		2.2	1.26-3.78	0.005	
*Age (ys)*	72/124								
<60		1.00	Ref	(0.04)		1.00	Ref	(0.01)	
≥60		1.7	1.01-2.74	0.04		1.9	1.14-3.27	0.01	
***Disease specific survival^2^***									
*GEX phenotypes^4^*	48/119				45/112				
High-immune		1.00	Ref	(0.009)		1.00	Ref	(0.06)	Gender, age, metastasis type
Pigmentation		1.7	0.83-3.28	0.2		1.4	0.71-2.92	0.3	
Proliferative		3.5	1.56-7.80	0.002		2.8	1.19-6.65	0.02	
*Gender*	51/125								
Female		1.00	Ref	(0.02)		1.00	Ref	(0.007)	
Male		2.2	1.13-4.11	0.02		2.8	1.34-5.90	0.006	
*Age (ys)*	51/124								
<60		1.00	Ref	(0.05)		1.00	Ref	(0.06)	
≥60		1.8	1.01-3.34	0.05		1.8	0.97-3.49	0.06	
*Metastasis type*	48/118								
In-transit		1.00	Ref	(0.03)		1.00	Ref	(0.3)	
Regional		0.39	0.16-0.92	0.03		0.63	0.25-1.56	0.3	

Abbreviations: CI, confidence interval.

1 Follow up starts at disease progression and ends at distant metastasis occurrence (=event).

2 Follow up starts at disease progression and ends at melanoma-specific death (=event).

3 The following confounders were included in the model: Gender, age (dichotomized at 60 years), and metastasis type (in-transit and regional). The confounders were selected based on their significance from the univariable analysis with P≤ 0.05.

4 Not including unclassified observations in the analysis.

* P-values for the pairwise comparisons were calculated using the Wald-test. Overall P-values (also from the Wald-test) are given within the parentheses.

Based on the findings from the network analysis described above, we divided the patients into low or high activity groups for each of the five expression modules. Each module was analyzed separately and further linked to the gene expression phenotypes (Figure S1C). Notably, the pigmentation phenotype could be split into low and high cell cycle activity groups with the high activity group being associated with a poor 5-year DMFS (HR, 2.6; 95% CI, 1.16-5.70; *P*=0.02, Figure 3D). The other phenotypes could also be further stratified, however, the number of patients included was too few to obtain statistical significance.

### Gene expression phenotypes and prediction of benefit from molecular targeted therapies

Since these molecular groupings exhibit an impact on survival, it may be hypothesized that the profiles could also correlate with therapeutic response. We thus evaluated whether the gene expression phenotypes were associated with response to therapies (MAPK pathway inhibition or MAGE-A3 vaccine) using three publicly available gene expression datasets.

**Figure 4:**
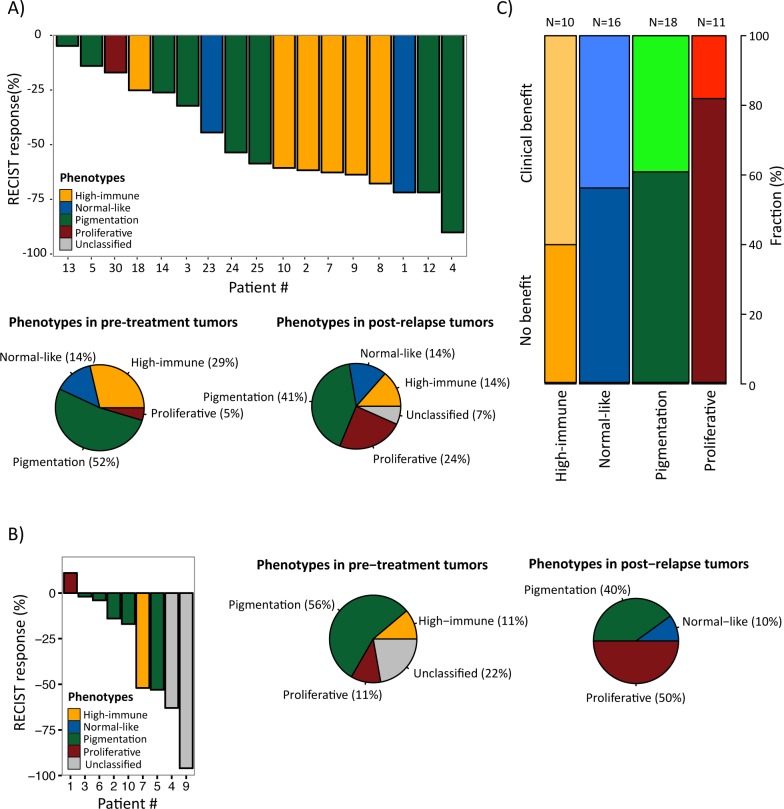
Gene expression phenotypes and prediction of clinical benefit from molecular targeted therapies **A**) Melanoma tumors from patients treated with BRAFi [16] or **B**) BRAFi/MEKi [17] were classified into the gene expression phenotypes and further analyzed for objective response (RECIST response, %) (4A, upper panel; 4B left panel) and phenotype distribution in pre-treatment and post-relapse biopsies (4A, lower panel; 4B, middle and right panel). **C**) Gene expression phenotype distribution in patients treated with MAGE-A3 vaccine [14]. The fraction of patients with clinical and no benefit is indicated for each phenotype.

In the first dataset (GSE50509, Rizos et al.), 21 patients with *BRAF*-mutant metastatic melanoma were treated with BRAF inhibitors (BRAFi, dabrafenib or vemurafenib) and evaluated for best objective response (RECIST response, %) and progression-free survival (PFS) [16], followed by gene expression profiling of tumor samples taken pre-treatment and post-relapse. When we classified this dataset into the gene expression phenotypes, we found no clear correlation between the clinical response and the pre-treatment phenotypes owing to small numbers (Figure 4A, upper panel). However, the single MITF-low proliferative sample responded poorly to treatment (RECIST response, %) with only 15 weeks until the patient progressed (patient 30). In contrast, the high-immune response-classified samples had a superior response with more than 25 weeks before progression (except in one case, patient 18) (Figure 4A, upper panel). Interestingly, the phenotype distribution was different in post-relapse samples, with an increased prevalence of MITF-low proliferative-classified cases (*P* = 0.06, Fisher's exact test; proliferative versus non-proliferative cases, excluding unclassified cases) (Figure 4A, lower panel).

The second dataset (GSE61992, Long et al.) included 10 patients with *BRAF*-mutant metastatic melanoma that were treated with a combination of dabrafenib and the MEK inhibitor (MEKi) trametinib and evaluated for RECIST response and PFS [17]. There were too few observations to draw any definitive conclusions, however again, the single MITF-low proliferative sample responded poorly to treatment (RECIST response, %) with only 2.8 months until the patient progressed (patient 1) (Figure 4B, left panel). In line with the results from the Rizos et al. dataset, there was an increased prevalence of MITF-low proliferative-classified cases after treatment, although not reaching significance (*P* = 0.3, Fisher's exact test; proliferative versus non-proliferative cases, excluding unclassified cases) (Figure 4B, middle and right panel). When combining the gene expression-based classification results from the datasets by Rizos et al. and Long et al. [16, 17], in total including 31 patients, the prevalence of MITF-low proliferative-classified cases increased after resistance emerged to either BRAFi alone or in a combination with BRAFi and MEKi (*P* = 0.02, Fisher's exact test; proliferative versus non-proliferative cases, excluding unclassified cases).

In the third study, we analyzed 56 patients that had been evaluated for response to MAGE-A3 immunotherapeutic treatment (22 with clinical benefit and 34 with no benefit) [14]. When we classified these samples into the gene expression phenotypes and analyzed the patients' treatment response to MAGE-A3, only 2/11 patients in the MITF-low proliferative phenotype had clinical benefit from treatment, while the highest proportion of responders was found in the high-immune response group (6/10) although not reaching statistical significance (*P*=0.2, Fisher's exact test of proliferative versus non-proliferative cases) (Figure 4C).

In summary, we provide initial indications that gene expression based classification may predict clinical benefit from targeted therapy.

## DISCUSSION

It is well established that melanoma can be genetically classified according to somatic gene mutation status. Although this stratification is relevant, controversy exists regarding the prognostic significance of classifying melanomas based only on *BRAF* and *NRAS* mutations [18, 19]. Gene expression profiling may provide additional information to current prognostic assessment in melanoma and several studies have associated an immune response gene signature with improved prognosis [5-7, 9].

Prognostic assessment of stage III melanoma is currently performed by histopathological characterization, determining the number of involved lymph nodes and the size of nodal metastatic disease. However these features do not capture the extent of heterogeneity present in this group of patients. Previously, we identified gene expression phenotypes reflecting biological mechanisms relevant in melanoma such as melanocyte differentiation, DNA repair and immunological responses [9]. The existence of the reported gene expression phenotypes was further supported by analysis of a large cohort of primary melanomas [10]. Moreover, Nsengimana et al. recently confirmed the independent prognostic significance of the gene expression phenotypes in a population-based British cohort of melanoma patients (Nsengimana et al. Accepted for publication in Oncotarget, 2015). In the current study, we demonstrate that the gene expression phenotypes hold prognostic information also in a regional metastatic setting and that patients with tumors classified as high-immune response have an improved survival outcome as compared to patients with pigmentation or proliferative classified tumors. The TCGA data further supported the significance of the gene expression phenotypes in metastatic melanoma. Importantly, gene expression classification adds prognostic information to conventional markers such as gender and age in regional metastatic melanoma patients. In all, this highlights that gene expression-based classification may improve prognostic stratification in metastatic melanoma.

Previously, large screening efforts have uncovered novel melanoma driver genes using whole-exome sequencing [20, 21]. Confirming these studies, we found *BRAF* and *NRAS* mutations as almost mutually exclusive genetic events and enrichment of *NF1* and *KIT* alterations in melanomas wild-type for *BRAF* and *NRAS*. Furthermore, we found alterations in *CDKN2A*, *CDK4*, *CCND1* and *RB1* to be almost mutually exclusive genetic events. In all, this suggests that there may be multiple ways of activating or inactivating certain pathways in melanoma. In this study, we investigated known cancer genes previously reported as mutated in melanoma within the context of the gene expression phenotypes. Overall, the most frequently mutated genes in our study (*BRAF*, *NRAS*, *TP53* and *PTEN*) were mutated at similar frequencies across the gene expression phenotypes. However, *CTNNB1* mutation was preferentially mutated in the pigmentation phenotype supporting the role of the Wnt/beta-catenin in activating MITF [22]. Thus, integrating mutation profiles with gene expression classification may contribute to understanding of the molecular composition of individual melanomas.

Importantly, *BRAF* mutation (V600E) is a predictive marker of BRAF inhibitor treatment and the majority of patients receiving such therapy have a dramatic initial response [23]. However, resistance eventually develops in a substantial fraction of the patients and several molecular mechanisms explaining this have emerged during the last few years [24, 25]. In the present study, we found an enrichment of MITF-low proliferative-classified melanoma tumors in the resistant fraction obtained after BRAFi or BRAFi/MEKi treatment. These results are in line with two recent studies showing that the MITF-low state is associated with an intrinsic resistance to MAPK pathway inhibition, as well as with an acquired resistance observed later in initially responding melanomas [26, 27]. The underlying mechanism for the development of resistance is not fully known, although melanoma tumors with low levels or absence of MITF have proliferative and invasive capacity that is independent of the MAPK signaling pathway. MITF is a melanocyte differentiation transcription factor considered to be the master regulator in pigmentation, but has also been described as a lineage-specific oncogene in melanoma [28, 29]. In all, this highlights the complexity of MITF function and the need for further studies on melanoma tumor specimens obtained from MAPK pathway inhibitor treated patients to fully investigate the role of melanocyte differentiation gene programs (including that of *MITF*) in resistance development.

The observation that immune response gene signatures may be associated with improved survival outcome is intriguing when considering novel immunotherapies, such as anti-CTLA4 and anti-PD1 antibodies in melanoma. Such gene signatures may be correlated with benefit from immunotherapy and thus of direct clinical relevance. Indeed, we found that 60% of the patients with tumors classified as high-immune response exhibited clinical benefit from MAGE-A3 immunotherapy, while only 18% of the MITF-low proliferative classified tumors had clinical benefit from the vaccine treatment. Ulloa-Montoya et al. presented similar results in their own study suggesting a predictive immunogenic gene signature for MAGE-A3 immunotherapy [14]. Similar observations have been found in ipilimumab treated patients further suggesting an important role of the tumor microenvironment for improved immunotherapy response [30]. In contrast, Snyder et al. elegantly described a study on genomic prediction of response to CTLA-4 blockade [31]. In detail, mutation load based on somatic neoepitopes were able to discriminate patients benefitting from CTLA-4 blockade and those not benefitting. Thus, it is likely that this may also influence response to MAGE-A3 vaccine and more extensive prospective studies on immunotherapy-treated patients are needed to define a clinically useful test.

In summary, we demonstrate that melanoma gene expression phenotypes are highly prognostic for survival outcome. Our data also provide evidence that the MITF-low proliferative phenotype is more common in post-relapse cases suggesting that these cells may be selected for during the course of MAPK pathway inhibitor treatment. Furthermore, we delineated the mutational landscape in the gene expression phenotypes providing support that integration of molecular data contributes to the understanding of melanoma. Gene expression profiling as well as targeting deep sequencing is easily performed, and therefore these approaches provide novel and promising ways to improve prediction of patient prognosis as well as prediction of treatment response to molecular targeted therapies in melanoma.

## MATERIALS AND METHODS

### Patients

This study was approved by the Regional Ethics Committee at Lund University (Dnr. 191/2007 and 101/2013). The sample cohort, representing a population-based retrospective collection (*n*=219), was obtained at the Department of Surgery at Skåne University Hospital (Figure S3).

The CMM (*n*=214) cohort comprised a minor subset of primary melanoma tumors (*n*=16) and a larger fraction of metastases including regional metastases (*n*=139), distant metastases (*n*=23), local recurrences (*n*=11) and in-transit metastases (*n*=15). In a small subset of the samples we were lacking associated stage data and these samples were further entitled as not available (NA) (*n*=10). In general, local metastases were either cutaneous or subcutaneous (10/11), in-transit metastases were subcutaneous (13/15), regional metastases were typically found in lymph nodes (124/139), and distant metastases were found either subcutaneously (10/23) or in visceral areas (10/23). A summary of the patient characteristics is provided in Table 1. This is an independent study without sample overlap with earlier studies performed by our group [9, 10].

### Gene expression and somatic mutation profiling

Genome-wide expression profiling was performed using Illumina Human-HT12v4.0 BeadChip arrays by standard methods. Data from this study have been submitted to the NCBI Gene Expression Omnibus (GEO) database (GSE65904). Detailed descriptions of the procedures and data analysis steps are provided in the Supplementary Data. The centroids from Harbst et al. were used to classify the samples into the four identified melanoma phenotypes [10]. The data were analyzed for technical variations using principal component analysis (PCA), (Figure S4) [32]. In order to further describe the phenotypes and find highly connected genes in the cohort, we created a melanoma network as previously described [15]. A subset of the samples was further analyzed using immunohistochemistry. In addition, we performed targeted deep sequencing of 1697 cancer associated genes in 146 patients (having a matched blood sample) out of the 214 CMM patients, as previously described [33]. Mutation data was visualized using Oncoprinter [34, 35]. The gene expression phenotypes and their clinical relevance were evaluated in three independent external datasets obtained from GEO (GSE50509 [16]; GSE61992 [17]; GSE35640 [14]). Before we performed the classification of the external samples, we combined our dataset with the above external datasets (pairwise merging) and applied distance weighted discrimination (DWD), (Figure S5) [36].

TCGA RNAseqv2 level 3 data (release 3.1.14.0, 2015-01-28), comprising 20,501 genes from 472 primary and metastatic samples was downloaded in the form of normalized RSEM count estimates (‘*rsem.genes.normalized_results' files) from the TCGA data portal (https://tcga-data.nci.nih.gov/tcga/).

## SUPPLEMENTARY MATERIALS AND METHODS, FIGURES AND TABLES


