# Supplementary material for: Molecular stratification of metastatic melanoma using gene expression profiling : Prediction of survival outcome and benefit from molecular targeted therapy
Source: Oncotarget. 2015 Mar 26;6(14):12297–309. doi: 10.18632/oncotarget.3655 (PMC4494939; doi:10.18632/oncotarget.3655)
Supplement: Supplementary file 1 [file oncotarget-06-12297-s001.pdf]

Molecular stratification of metastatic melanoma using gene expression profiling - prediction of survival outcome and benefit from molecular targeted therapy

SUPPLEMENTARY MATERIALS AND METHODS

Tumor specimens and RNA isolation

Tissues from melanoma tumors (*n* = 219) were obtained from the Department of Oncology at Lund University. After surgical removal of the tumors, all biopsies were snap frozen in liquid nitrogen and further stored at ultra-low temperature for later experimental use. Frozen tissues were homogenized using a TissueLyser (Qiagen) and DNA and RNA extracts were isolated using the AllPrep kit (Qiagen). To confirm good RNA quality, all extracts were analyzed on an Agilent Bioanalyzer 2100 (Agilent) and only samples with a RIN value larger than 6 was included in the study. DNA from blood samples was extracted using the DNeasy Blood and Tissue kit (Qiagen).

Preparation and hybridization

Total RNA was converted to cDNA, biotin-labeled cRNA and finally hybridized to 50-mer probes on HumanHT-12 v4.0 Expression BeadChips (Illumina) according to Illumina's assay guide for Direct Hybridization Whole-Genome Expression assays. After washing and staining steps, the BeadChips (12 arrays of 47323 probes in each) were scanned using iScan.

Gene expression analysis and profiling

Initial preprocessing of the data was executed using the GenomeStudio software (Illumina), which included removal of outlier beads and calculation of average bead signals and detection p-values. Data normalization was performed using the algorithm for cubic spline quantile-normalization [1]. Further processing was done using the R statistical software. Briefly, the data was log2 transformed and probes with a detection

p-value <0.01 were kept if present in minimum 80% of the samples. Of the 13955 probes retained, 13744 probes corresponded to RefSeq features. These RefSeq features mapped to 11051 unique genes and the most varying probe for each gene was kept in the data. In order to compare gene expression levels across samples, the 11051 probes were mean centered. The centroids from Harbst et al. was used to classify the samples into any of the four recently identify melanoma subtypes [2]. In detail, the nearest centroid classification was used to classify a sample according to the maximum correlation of the sample with the four centroids (Pearson's correlation > 0.2, otherwise set as "unclassified").

Network construction and module expression

A gene co-expression network was created based on correlations between the genes in the data set and five modules of highly connected genes were extracted [3]. Briefly, the initial network was created by connecting nodes (genes) by edges (representing correlations) using a correlation cutoff of 0.6 and only including genes with five or more neighbors. Based on gene ontology analysis and published associations with melanoma-specific tumor biology, the five modules were further entitled as: the micropthalmia-associated transcription factor (MITF) module, including genes such as *MITF, MLANA*, *SILV*, *CDK2* and *ETV5*; the cell cycle module, containing genes related to the M phase, such as *CCNB2* and *CENPF*; the stroma module consisting of extracellular matrix-related genes; the immune response module representative of genes enrolled in tumor infiltrating lymphocyte and antigen presentation; and finally also an interferon module consisting of genes responsive of interferon treatment. The resulting gene expression landscape consisting of 394 genes scattered around the five core modules was visualized in Cytoscape (Figure 1B) [4]. Module activity scores were

calculated for all samples, *i.e.* mean expression values of all genes representing the particular modules.

SureSelect deep targeting sequencing

Target enrichment design, library preparation and data processing were performed as previously described [5]. Briefly, 1697 frequently mutated cancer-associated genes were selected based on information in the COSMIC database and in the literature to create the SureSelect target enrichment design (5.5 MB coverage by 120 bp-long tiling probes). DNA samples were sheared, end-repaired and given a unique adapter (sample specific barcode) before PCR-amplification. The adapter-ligated fragments were subjected to target enrichment (SureSelect, Agilent), PCR-amplification and sequencing according to Illumina Paired-End Sequencing Library Protocol on a HighSeq2000.

Principal component analysis (PCA)

PCA was used to assess that the variation in the gene expression data was due biological factors and not systematic experimental artifacts. Briefly, PCA functions by reducing the dimensionality of the data by extracting principal components (PCs), *i.e.* uncorrelated linear vectors further explaining the variation in the data [6]. The PCs are then tested for association with the biological and technical variables to elucidate their impact on the variation in the data. We performed PCA by using the *swamp* package in R [7]. Results are shown as a heatmap of the associations between PCs and sample annotations (Figure S4).

Immunohistochemistry (IHC)

A subset of the melanoma tumors (n = 59), representing the four major gene expression phenotypes, was selected for immunohistochemistry. After formalin-fixed paraffin-embedding, sections (4 µm) were captured and prepared according to standard procedures. Staining was performed using antibodies against MITF (clone: C5, Thermo Fisher), cluster of differentiation 3 (CD3; polyclonal, DAKO) and Ki67 (clone: MIB-1, DAKO), together with the DAKO Envision horseradish peroxidase rabbit/mouse kit system and the Dakocytomation Autostainer (DAKO). Further more, sections were also stained with hematoxylin and eosin (HE) to see structural patterns in the tumor tissues.

Validation sets

TCGA RNAseqv2 level 3 data (release 3.1.14.0, 2015-01-28), comprising 20,501 genes from 472 primary and metastatic samples (barcodes '01' and '06'), was downloaded in the form of normalized RSEM count estimates ('*rsem.genes.normalized_results' files). The data was quantile-normalized between samples using limma [8], given an offset of 32, capped at 65,000, log-transformed and median-centered over genes. As described in the above section "*Gene expression analysis and profiling*", samples were classified into any of the four melanoma subtypes using the nearest centroid classification (Pearson's correlation > 0.1, otherwise set as "unclassified"). The 438 available centroid genes found in the data were monitored for batch effects using TCGA's technical annotations.

The gene expression phenotypes and their clinical relevance were evaluated in three independent external datasets obtained from the Gene Expression Ominbus (GEO) repository (GSE50509 [10]; GSE61992 [11]; GSE35640 [12]). In the first study, 21

patients were treated with the BRAF inhibitors (BRAFi) dabrafenib or vemurafenib and evaluated for best objective response (RECIST response, %), progression-free survival (PFS) and screened for resistance mechanisms [10]. In total, 50 specimens were being analyzed in this study including 21 pre-treatment tumors and 29 post-relapse tumors. In the second study, 10 patients were treated with a combination of BRAFi (dabrafenib) and the MEK inhibitor (MEKi) trametinib, and analyzed for similar endpoints as in the prior study. However, gene expression data was only available for 9 patients, thus including 19 specimens in total (9 pre-treatment tumors and 10 post-relapse tumors) [11]. In the third study, 56 patients were analyzed for a pre-treatment gene expression signature predictive of response to MAGE-A3 immunotherapeutic treatment and each patient were assigned a status of responder or non-responder [12].

Genome-wide gene expression microarray was performed using Illumina Human HT-12 v4 BeadChip arrays (Rizos' and Long's studies) and Affymetrix HG-U133.Plus 2.0 system (Ulloa-Montoya's study) [10, 12]. We performed similar preprocessing of the three already normalized datasets obtained from GEO including addition of a probe presence filter, log2 transformation and KNN-imputation (the latter step was only performed in Rizos' and Long's data). In detail, for samples run on the Illumina platform, we removed probes when less than 80% of the samples had a detection p-value < 0.01, whereas in the Affymetrix data the cutoff was set to 0.1.

Combining datasets using Distance Weighted Discrimination (DWD)

DWD is a well-known classification method, which has also proven to be very useful in microarray datasets to compensate for systematic biases [13, 14]. Based on the findings that molecular portraits are conserved across platforms [15], we combined our dataset with the above external datasets and applied DWD adjustment before

performing nearest centroid classification. Initially, all external datasets were preprocessed as described in the above section "*Validation sets*" and pairwise analyzes were performed between our dataset and the external datasets (pairwise merging). Briefly, common centroid genes across the itemed datasets were extracted (Our/Rizos, 260 genes; Our/Long, 281 genes; Our/Ulloa-Montoya, 220 genes). Individual datasets (genes not mean centered) were combined and adjusted in a pairwise manner using the *InSilicoMerging* package in R calling the "DWD" method. The genes in the pairwise combined and adjusted data were mean centered across all the data and further classified. In order to check for remaining source biases, the data were visualized after DWD adjustment in a multidimensional scaling (MDS) plot (Figure S5) but also analyzed using hierarchical clustering to conclude sample dispersion in a source independent manner (results not shown).

Statistics

All statistical analyzes were performed using R (two-sided tests). Fisher's exact test and Kruskal-Wallis test were performed to compare gene expression phenotypes with clinical characteristics. All survival analyses were made using the *survival* package in R. In addition, we performed non-parametric Kruskal-Wallis tests to analyze the module activity scores against clinical characteristics.

Supplement text references

1. Vallon-Christersson J, Nordborg N, Svensson M and Hakkinen J. BASE--2nd generation software for microarray data management and analysis. BMC bioinformatics. 2009; 10:330.
2. Harbst K, Staaf J, Lauss M, Karlsson A, Masback A, Johansson I, Bendahl PO, Vallon-Christersson J, Torngren T, Ekedahl H, Geisler J, Hoglund M, Ringner M, Lundgren L, Jirstrom K, Olsson H, et al. Molecular profiling reveals low- and high-grade forms of primary melanoma. Clin Cancer Res. 2012; 18(15):4026-4036.

3. Fredlund E, Staaf J, Rantala JK, Kallioniemi O, Borg A and Ringner M. The gene expression landscape of breast cancer is shaped by tumor protein p53 status and epithelial-mesenchymal transition. Breast Cancer Res. 2012; 14(4):R113.
4. Shannon P, Markiel A, Ozier O, Baliga NS, Wang JT, Ramage D, Amin N, Schwikowski B and Ideker T. Cytoscape: a software environment for integrated models of biomolecular interaction networks. Genome research. 2003; 13(11):2498-2504.
5. Harbst K, Lauss M, Cirenajwis H, Winter C, Howlin J, Torngren T, Kvist A, Nodin B, Olsson E, Hakkinen J, Jirstrom K, Staaf J, Lundgren L, Olsson H, Ingvar C, Gruvberger-Saal SK, et al. Molecular and genetic diversity in the metastatic process of melanoma. The Journal of pathology. 2014; 233(1):39-50.
6. Ringner M. What is principal component analysis? Nature biotechnology. 2008; 26(3):303-304.
7. Lauss M, Visne I, Kriegner A, Ringner M, Jonsson G and Hoglund M. Monitoring of technical variation in quantitative high-throughput datasets. Cancer informatics. 2013; 12:193-201.
8. Smyth GK. Linear models and empirical bayes methods for assessing differential expression in microarray experiments. Statistical applications in genetics and molecular biology. 2004; 3:Article3.
9. Jonsson G, Busch C, Knappskog S, Geisler J, Miletic H, Ringner M, Lillehaug JR, Borg A and Lonning PE. Gene expression profiling-based identification of molecular subtypes in stage IV melanomas with different clinical outcome. Clin Cancer Res. 2010; 16(13):3356-3367.
10. Rizos H, Menzies AM, Pupo GM, Carlino MS, Fung C, Hyman J, Haydu LE, Mijatov B, Becker TM, Boyd SC, Howle J, Saw R, Thompson JF, Kefford RF, Scolyer RA and Long GV. BRAF inhibitor resistance mechanisms in metastatic melanoma: spectrum and clinical impact. Clin Cancer Res. 2014; 20(7):1965-1977.
11. Long GV, Fung C, Menzies AM, Pupo GM, Carlino MS, Hyman J, Shahheydari H, Tembe V, Thompson JF, Saw RP, Howle J, Hayward NK, Johansson P, Scolyer RA, Kefford RF and Rizos H. Increased MAPK reactivation in early resistance to dabrafenib/trametinib combination therapy of BRAF-mutant metastatic melanoma. Nature communications. 2014; 5:5694.
12. Ulloa-Montoya F, Louahed J, Dizier B, Gruselle O, Spiessens B, Lehmann FF, Suciu S, Kruit WH, Eggermont AM, Vansteenkiste J and Brichard VG. Predictive gene signature in MAGE-A3 antigen-specific cancer immunotherapy. J Clin Oncol. 2013; 31(19):2388-2395.
13. Benito M, Parker J, Du Q, Wu J, Xiang D, Perou CM and Marron JS. Adjustment of systematic microarray data biases. Bioinformatics. 2004; 20(1):105-114.
14. Huang H, Lu X, Liu Y, Haaland P and Marron JS. R/DWD: distance-weighted discrimination for classification, visualization and batch adjustment. Bioinformatics. 2012; 28(8):1182-1183.
15. Hu Z, Fan C, Oh DS, Marron JS, He X, Qaqish BF, Livasy C, Carey LA, Reynolds E, Dressler L, Nobel A, Parker J, Ewend MG, Sawyer LR, Wu J, Liu Y, et al. The molecular portraits of breast tumors are conserved across microarray platforms. BMC genomics. 2006; 7:96.

Supplementary Table 1. Gene symbols of the five melanoma network modules.

Immune Response				Stroma		Cell cycle	MITF	Interferon
ACSL5	SLCO2B1	IDO1 PRD	TNFSF10 B	PCSK5		NEK2 AN	RAB17 GP	IFIT1 I
HLA-	AIM2 PAP	M1 DOCK2	TG2 ARHGA		ABI3BP CF	LN PRC1	R56 RAB3	FIT3 IF
DRB4 SRGN	LN GIMAP	NCKAP1L	P25 CD74 S	H		CCNB2 C	8 D4S234	I44 IFI
CXCL16 FC	2 ITGB2 C	HAVCR2 A	TAT1 DENN		PDPN PDL	DC20 MA	E IRF4 RO	T2 IFI2
N1	YSLTR1 F	IF1 PSMB9	D2D AMICA	IM1		D2L1 CE	PN1B ERB	7 IFI44
LST1 ARH	UCA1 RAS	FPR3	1		FNDC1 CO	NPF PBK	B3 ATP1A	L IFI6
GAP9 EPST	AL3 SLAM	IGJ KYN	ADAMDEC1	L6A2		CDC45L C	1 SLC7A5	
I1 HCG4 G	F6 IFI30 G	U CCL4L	SKAP1		CDH11 MF	KS2 NCA	ETV5 CHC	
VIN1	IMAP5 CX	2 IRF1	HLA-	AP5		PG	HD6 LYST	
C2 CCL4	CR4 PIK3	SLC1A3	DPA1 RARR		NUAK1 PO		MITF IGS	
L1	AP1 CFB	BASP1 C	ES3 GMFG	DN			F11 CAPN	
GLRX	CD52 CY	D2 CD38	SYK PPP1R		APLNR PR		3 MLANA	
HLA-	BB LBH		16B RASGR	ICKLE1			MLPH CD	
DMA FYB	HCST AB	CCDC109B	P1 SERPINA		F13A1 LR		K2	
HLA-	CG1 ALO	FLI1 PSCDB	1 RBM47 CC	RC32			PIR ROPN	
E MFNG	X5 GZMK	P CSF1R MS	ND2 APOBE		ECSCR EB		1 SNCA C	
GZMA FG	TRIM22	4A7 CD69	C3G C1QA G	F1			ABLES1 S	
R FGD2 B	CD37 LP	GBP2 G	IMAP6 GIM		COL3A1 C		LC45A2 P	
IRC3 FCG	XN	PR34	AP7 HLA-	D34			PM1H	
R1A NAP	PLCG2 CXC	CD7 CCL19	DQB1 HLA-		SMOC2 GU		SILV INP	
SB SLAM	L13 HLA-	CAMK1G FC	DPB1 TNFA	CY1A3			P4B	
F8	DQA1 PRKC	GR1B CD86	IP8 LY86		PLAC9 GL			
RFTN1 IL2R	B1 RAC2	TMEM176A	GBP4 D	T8D2				
B APOL3 CS	PLEK SP	TNFSF13B G	HRS9 L		EFNB2 EG			
T7 ARHGAP	INT2 EV	PR65 KLRB1	YZ	FLAM				
30 PIM2 CE	I2A LMO	IGLL1 SNOR	HLA-		FABP4 CO			
BPA HCP5 F	2 CD163	D13 CTSS IN	DOA PIK3I	L6A3				
ERMT3 SLA	TLR7 H	PP5D	P1 FOLR2		CXCR7 TC			
APBB1IP LI	LA-B	EVL HCLS	GBP5	F4				
ME1 DOCK8	RARRES1	1 GIMAP1	SPI1 CCL		CD36 C1Q			
CRKRS ITGA	GIMAP4 CD	IGLL3 CD	2 MS4A6	TNF5				
X SIGLEC14	8A SERPIN	53 CD3D	A CD79B		CCL14 C1			
PLA2G7 RA	G1 HLA-	FCER1G G	GLIPR1 ST	R				
SGRP3 PTG	DRB3 GLIP	BP1 MS4	X11 EBI2		COLEC12			
ER4 GRAP L	R2 TNFRSF	A4A PTPN	ARHGDIB I	PALMD				
APTM5 IKZ	1B LTB LA	6 CD79A	L32 TBXA		PODXL IG			
F1 CD247 G	MP3 FGD3	EVI2B SE	S1 CCL5 M	FBP6				
ZMB PIK3C	FGL2 ST	L1L3 IGSF	AP4K1 CT		GPX3 COL			
G CORO1A C	AT4 GIM	6 SLC15A	SH	1A2				
LEC4A LAT2	AP8 IL7R	3 NKG7 C	HLA-		LRIG1 IGF			
		D48 MMP	DRA PSCD	BP4				
BTK CECR	KCNMA1	9 C1QC LC	4 ALOX5A		MXRA5 CR			
1 C1QB PR	HLA-	P1 SLC40	P SEMA4D	ISPLD2				
KCH ECGF	DMB SPOC	A1 LYN			LEPR COL			
1 SIGLEC1	K2 SLC7A7	HLA-F	SH2D1A	12A1				
0 AKNA IR	CXCL9 CD				CD248 CO			
F8	14 ICAM2			L15A1				
	NCF1 ITM				PDGFRL S			
	2A IL18BP			PARC				
					SULF2 EP			
				AS1				
					COL5A2 D			
				CN	F3			
				BGN				
					CLEC3B FB			
				LN1				
					CDH5 SFR			
				P2				
					OLFML2B			
				COL1A1				

Supplementary Table 2. Gene expression phenotype-specific genetic events.

Genetic events		High-immune group	Pigmentation group	Proliferative group
			P*	
Gene	***Alteration***			
BRAF	mut	0.5	0.9	0.3
NRAS	mut	0.4	0.6	1
NF1	mut	1	0.7	1
KIT	mut/amp	1	0.4	0.6
CDKN2A	mut/del m	0.2	0.4	0.05
TP53	ut mut/de	1	0.6	0.4
PTEN	l mut	0.6	1	0.4
PIK3CA	mut mut	1	0.1	0.1
CTNNB1	mut/amp	0.1	0.02	0.7
RB1	amp	0.2	0.1	1
CDK4	amp	1	0.7	1
CCND1	amp	0.5	0.04	0.5
MITF		0.3	0.04	0.6
NOTCH2		1	0.7	1

* P-
values shown in the table were calculated using Fisher's exact test. Each gene expr
ession phenotype was compared to a combined group of all other samples (excluding u
nclassified samples), n = 137 samples in the analysis.

Supplementary Table 3. Survival outcome analysis in patients with regional metastatic disease; univariable Cox regression analysis of clinical parameters.

	Disease-specific survival[1]				Distant metastasis-free survival[2]			
**Clinical parameters**	**Events/*N***	**HR**	**95% CI**	***P****	**Events/*N***	**HR**	**95% CI**	***P****
Metastasis								
Gender	51/125				72/125			
Female		1.00	Ref	(0.02)		1.00	Ref	(0.02)
Male		2.2	1.13–4.11	0.02		1.9	1.13–3.17	0.02
Age (ys)	51/124				72/124			
<60		1.00	Ref	(0.05)		1.00	Ref	(0.04)
≥60		1.8	1.01–3.34	0.05		1.7	1.01–2.74	0.04
Metastasis type	48/118				68/118			
In-transit		1.00	Ref	(0.03)		1.00	Ref	(0.08)
Regional		0.39	0.16–0.92	0.03		0.52	0.25–1.09	0.08
Site of metastasis	46/114				66/114			
Subcutaneous		1.00	Ref	(0.3)		1.00	Ref	(0.06)
Lymph node		0.64	0.25–1.62	0.3		0.51	0.25–1.03	0.06
Affected nodes	34/88				47/88			
≤1		1.00	Ref	(0.2)		1.00	Ref	(0.4)
1<		1.6	0.79–3.04	0.2		1.3	0.71–2.27	0.4
Previous primary tumor[2]								
Age (ys)	46/105				64/105			
<60		1.00	Ref	(0.2)		1.00	Ref	(0.2)
≥60		1.5	0.80–2.63	0.2		1.4	0.86–2.35	0.2
Breslow thickness (mm)	45/101				63/101			
≤1.0		1.00	Ref	(0.6)		1.00	Ref	(0.8)
1.01–2.0		0.63	0.24–1.62	0.3		0.88	0.38–2.04	0.8
2.01≤4.0		1.02	0.42–2.49	1.0		0.87	0.38–2.00	0.7
4.0<		0.75	0.29–1.94	0.6		1.2	0.52–2.67	0.7
Clark level	36/81				53/81			
III		1.00	Ref	(0.8)		1.00	Ref	(0.8)
IV		0.91	0.46–1.81	0.8		1.1	0.61–1.95	0.8
Histologic type	37/88				53/88			
Unknown primary		1.00	Ref	(0.2)		1.00	Ref	(0.3)
SSM		2.2	0.73–6.45	0.2		2.2	0.80–5.78	0.1
NM		1.3	0.44–3.97	0.6		2.0	0.78–5.25	0. 1
Primary site	44/98				61/98			
Trunk		1.00	Ref	(0.2)		1.00	Ref	(0.5)
Upper limbs		0.58	0.24–1.42	0.2		0.63	0.29–1.37	0.2
Lower limbs		0.57	0.29–1.11	0.1		0.83	0.48–1.43	0. 5
Ulceration	18/50				30/50			
No		1.00	Ref	(0.5)		1.00	Ref	(0.3)
Yes		1.4	0.54–3.59	0.5		0.71	0.35–1.45	0.3

Abbreviations: NA, not available; SSM, superficial spreading melanoma; NM, nodular melanoma; CI, confidence interval; HR, hazard ratio.

1 Follow up starts at disease progression and ends at melanoma-specific death (=event).

2 Follow up starts at disease progression and ends at distant metastasis occurrence (=event).

* P-values for the pairwise comparisons were calculated using the Wald-test. Overall P-values (also from the Wald-test) are given within the parentheses.

A

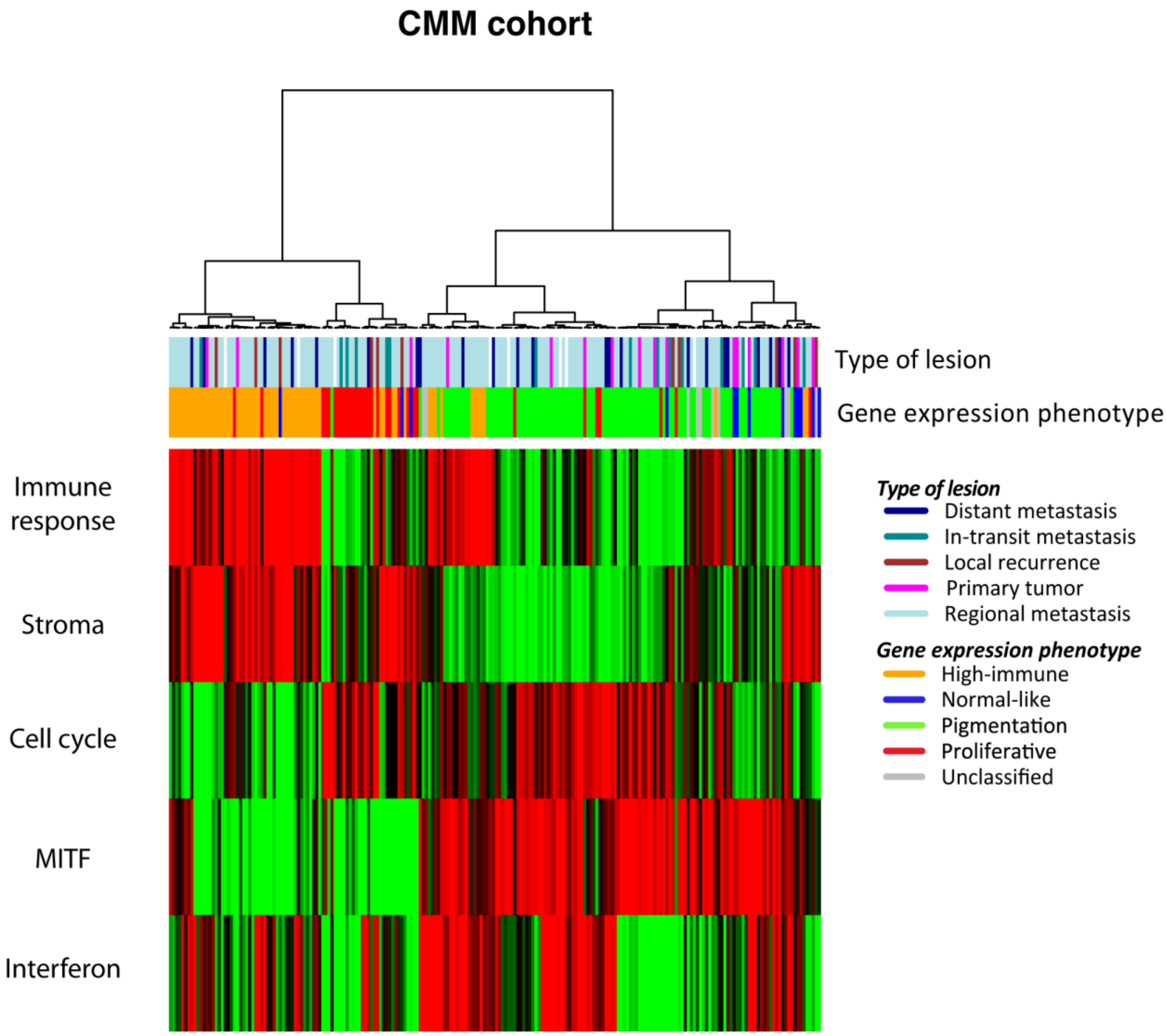
CMM cohort
Type of lesion
Gene expression phenotype
Immune response
Stroma
Cell cycle
MITF
Interferon
Type of lesion
Distant metastasis
In-transit metastasis
Local recurrence
Primary tumor
Regional metastasis
Gene expression phenotype
High-immune
Normal-like
Pigmentation
Proliferative
Unclassified

B

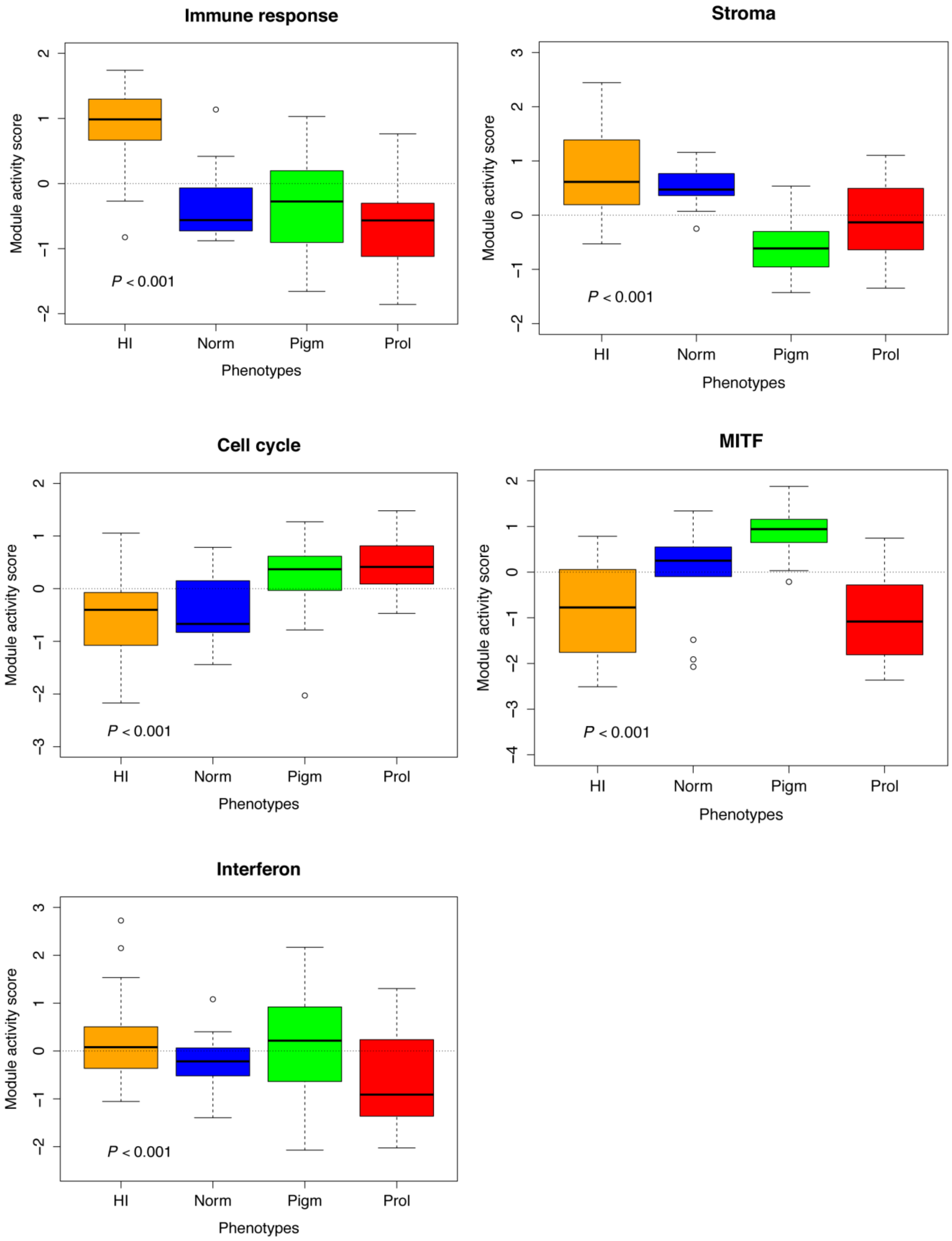
Immune response
Module activity score
P < 0.001
HI
Norm
Pigm
Prol
Phenotypes
Stroma
Module activity score
P < 0.001
HI
Norm
Pigm
Prol
Phenotypes
Cell cycle
Module activity score
P < 0.001
HI
Norm
Pigm
Prol
Phenotypes
MITF
Module activity score
P < 0.001
HI
Norm
Pigm
Prol
Phenotypes
Interferon
Module activity score
P < 0.001
HI
Norm
Pigm
Prol
Phenotypes

C

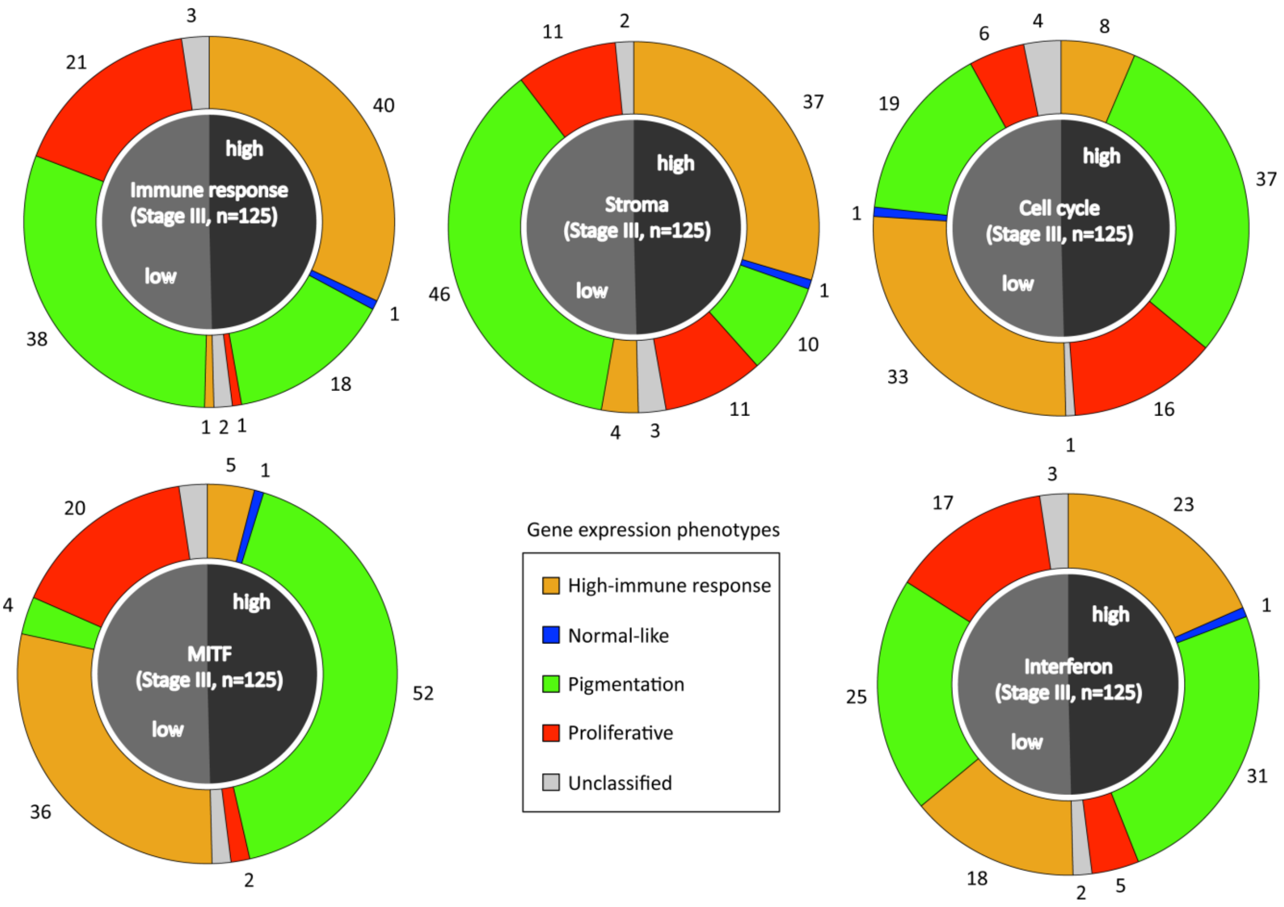

Supplementary Figure 1

Correlation of the gene expression phenotypes with the network modules. A) Heatmap of tumor-specific module activity scores (rows) and samples (columns) ordered according to the gene expression phenotypes. Color bars indicate gene expression phenotypes and type of tumor lesion. B) Depiction of median module activity scores stratified by gene expression phenotype. C) Low and high network module groups and their association with the gene expression phenotypes.

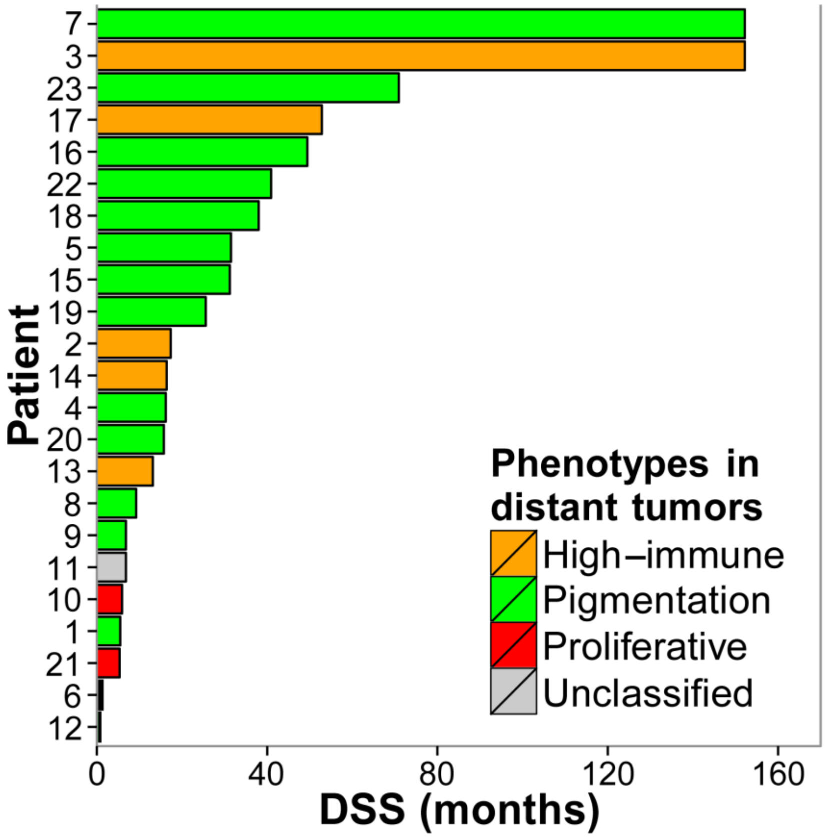

Supplementary Figure 2

Correlation of disease-specific survival (DSS) and assigned gene expression phenotypes in 23 stage IV melanoma patients with distantly spread metastases.

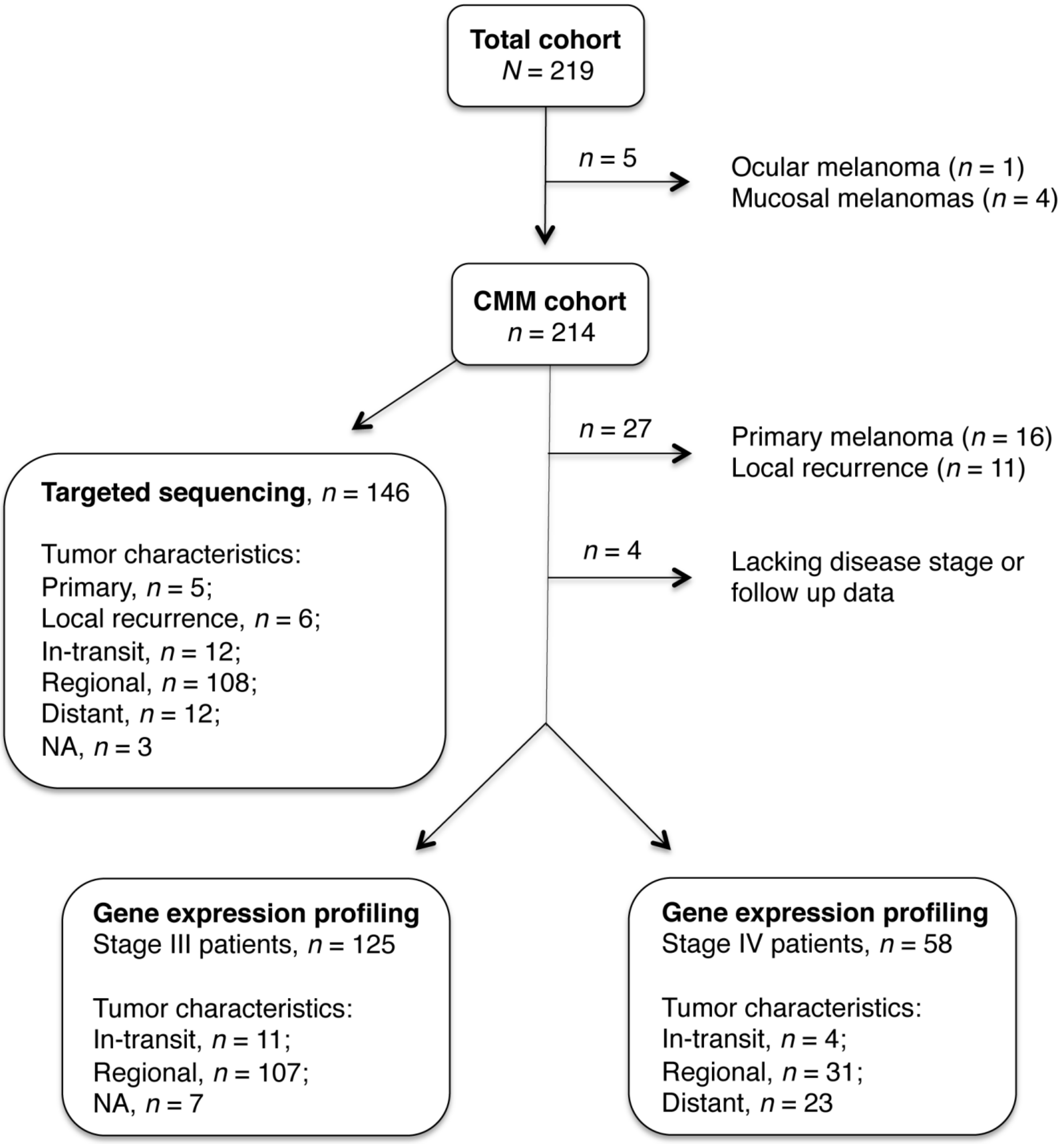

Supplementary Figure 3

Consort diagram for melanoma patients included in the present study. In total 219 melanoma specimens were subjected to gene expression profiling, whereas only the cutaneous subset, *n*=214, was considered in subsequent analyzes. Deep targeted sequencing was performed on 146 melanoma samples.

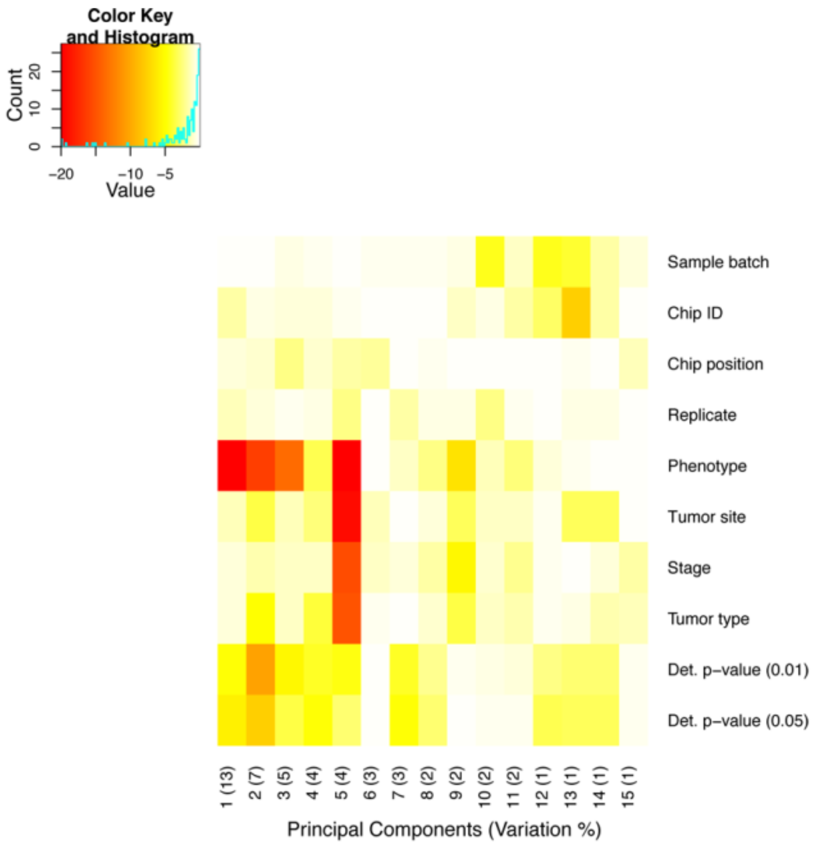

Supplementary Figure 4

PCA was applied to control that the variation in the gene expression data was due biological factors and not systematic experimental artifacts. Samples were projected onto PCs (top 15 PCs) and further correlated to the sample annotations. Technical factors to be evaluated included sample batch (plate), chip id (hybridization batch), chip position and any differences found among the biological replicates. Biological and clinical factors included gene expression phenotypes, tumor site, disease stage, tumor type and the number of active genes as counted by probes with detection p-values (Det. p-value) less than 0.01 or 0.05 for each sample.

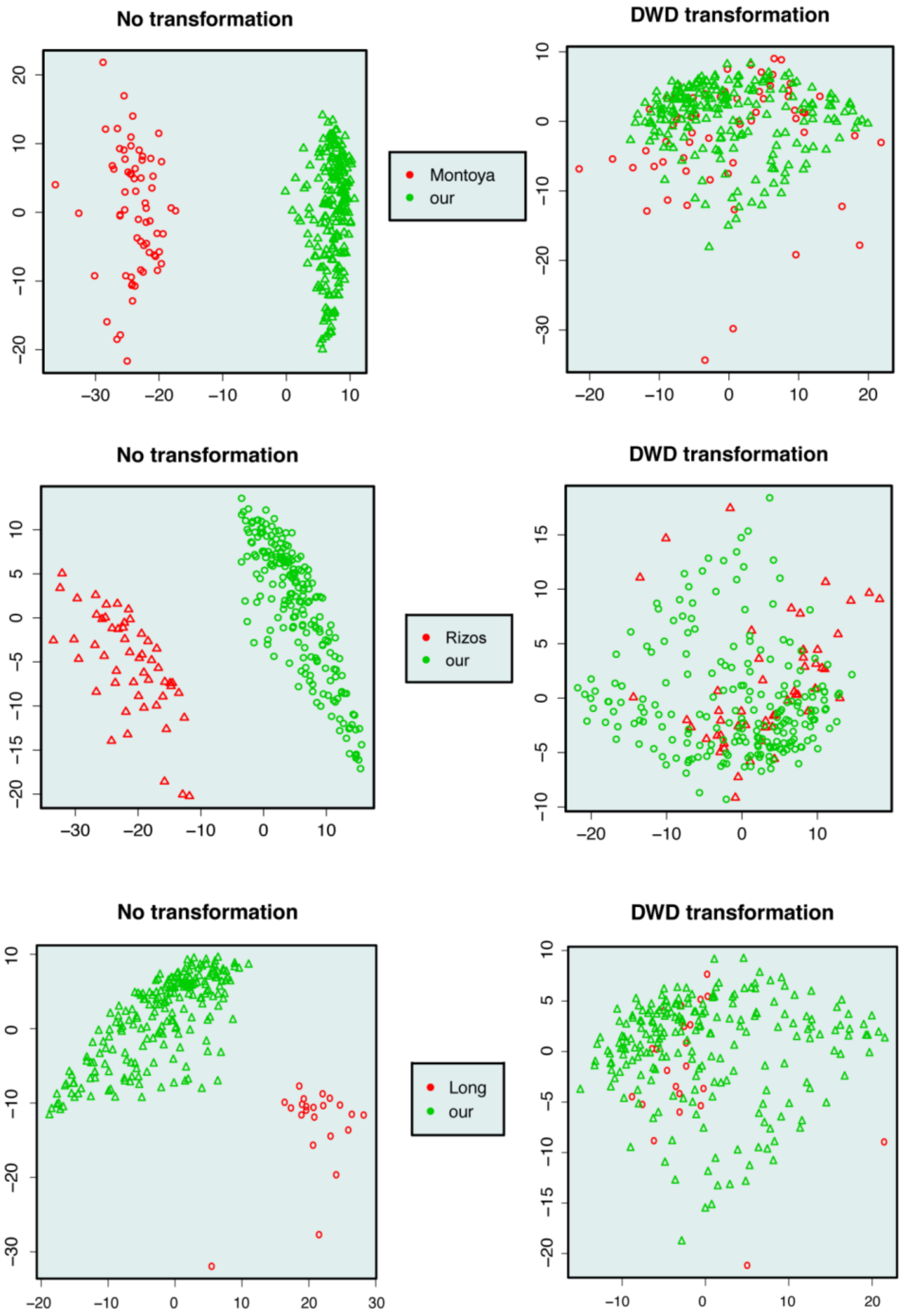

Supplementary Figure 5

Visualization of potential sample source bias after pairwise merging of datasets using inSilicoMerging (R package) calling DWD adjustment. The DWD adjustment was performed using common centroid genes across the itemed datasets (Our/Rizos, 260 genes; Our/Long, 281 genes; Our/Ulloa-Montoya: 220 genes).
